# Sexuality in autism spectrum disorders– case series

**DOI:** 10.1192/j.eurpsy.2026.10289

**Published:** 2026-07-31

**Authors:** V. Mandic Maravic

**Affiliations:** 1 Institute of Mental Health; 2 Faculty of Medicine, University of Belgrade, Belgrade, Serbia

## Abstract

**Abstract:**

Sexuality is a complex aspect of human functioning, including gender identity, roles, sexual orientation, pleasure, intimacy and reproduction. In the past decades, the view of sexuality in persons with ASD has developed from being „asexual” to experiencing intense romantic and sexual needs. During the development of sexuality, persons with ASD are often without adequate education, as parents often feel incompetent to educate their children. Also, specific sensory sensitivity alters the modality of sexual contact in persons with ASD. General self-awareness in ASD is lower, and it is an important aspect of sexuality. There is increasing evidence of specifics in sexuality and sexual orientation in persons with ASD, with non-heterosexual orientation being significantly more prevalent than in the general population, especially in females with autism. Gender dysphoria is present in higher prevalence in persons with ASD than in the general population. Individuals with ASD have higher prevalence of mental health problems, especially if they are a part of sexual and gender minorities. This case-based presentation aims to emphasize the importance of sexuality in ASD, an often disregarded subject in this population.

**Disclosure of Interest:**

None Declared

